# Age-related changes of cholestanol and lathosterol plasma concentrations: an explorative study

**DOI:** 10.1186/s12944-019-1176-3

**Published:** 2019-12-30

**Authors:** Monica Gelzo, Maria Donata Di Taranto, Concetta Sica, Antonio Boscia, Francesco Papagni, Giuliana Fortunato, Gaetano Corso, Antonio Dello Russo

**Affiliations:** 1 Department of Molecular Medicine and Medical Biotechnologies, University of Naples Federico II, Via Sergio Pansini 5, 80131 Naples, Italy; 2 CEINGE Biotecnologie Avanzate s.c. a r.l., Via Gaetano Salvatore 486, 80145 Naples, Italy; 3 Department of Clinical and Experimental Medicine, University of Foggia, Viale L. Pinto 1, 71122 Foggia, Italy

**Keywords:** Cerebrotendinous xanthomatosis, Lathosterolosis, Plasma sterol profile, Gas chromatography

## Abstract

**Background:**

Cerebrotendinous xanthomatosis (CTX) and Lathosterolosis represent two treatable inherited disorders of cholesterol metabolism that are characterized by the accumulation of cholestanol and lathosterol, respectively. The age of the patients suspected of having these disorders is highly variable due to the very different phenotypes. The early diagnosis of these disorders is important because specific therapeutic treatment could prevent the disease progression. The biochemical diagnosis of these defects is generally performed analyzing the sterol profile.

Since age-related levels of these sterols are lacking, this study aims to determine a preliminary comparison of plasma levels of cholestanol and lathosterol among Italian unaffected newborns, children and healthy adults.

**Methods:**

The sterols were extracted from 130 plasma samples (24 newborns, 33 children and 73 adults) by a liquid-liquid separation method and quantified by gas chromatography coupled with a flame ionization detector.

**Results:**

Cholesterol, cholestanol and lathosterol levels together with the cholestanol/cholesterol and lathosterol/cholesterol ratios are statistically different among the three groups. Cholesterol levels progressively increased from newborns to children and to adults, whereas cholestanol/cholesterol and cholestanol/lathosterol ratios progressively decreased from newborns to children and to adults. Lathosterol levels were higher in adults than in both newborns and children. In the total population a positive correlation was observed between cholesterol levels and both cholestanol (correlation coefficient = 0.290, *p* = 0.001) and lathosterol levels (correlation coefficient = 0.353, *p* <  0.0001).

**Conclusions:**

Although this study can only be considered an explorative experience due to the low number of analyzed samples, we revealed several differences of plasma cholestanol and lathosterol levels and their ratios to cholesterol levels among newborns, children and adults. These evidences indicate the need of age-related reference values of cholestanol and lathosterol concentrations, including also newborns and children.

## Introduction

The inherited metabolic disorders of cholesterol synthesis and catabolism are characterized by an overall breadth and diversity of clinical features including major somatic and/or CNS malformations and dysmorphic features [1]. Although these disorders show overlapping phenotypes, they are characterized by the accumulation of specific non–cholesterol sterols in blood and tissues of affected patients [2, 3]. In addition, even though some cases are reported as normocholesterolemic, generally the alteration of plasma cholesterol concentration and particularly the non-cholesterol sterol/cholesterol ratio is suggestive of a cholesterol metabolism defect [4]. Hence, an accurate analysis for the identification and quantification of these metabolites are essential to address the diagnostic process of these defects. A useful biomarker is cholestanol, an intermediate of bile acid synthesis that derives primarily from the catabolism of cholesterol in the liver [5, 6]. Increased cholestanol concentration is found in plasma and tissues of subjects affected by CTX, (OMIM #213700), an autosomal recessive disorder due to mutations in the *CYP27A1* gene (OMIM *606530) resulting in deficiency of sterol-27-hydroxylase and a sharp decrease in chenodeoxycholic acid [7, 8]. The most common clinical features of CTX are diarrhea, cataracts, tendon xanthomas, and neurological manifestations [9]. The incidence of CTX in Italy was estimated between 1.4 and 3.9 cases: 1000000 per year (http://old.iss.it/publ/).

Beside the cholestanol, plasma sterol profile of CTX patients shows increased concentrations of biosynthesis cholesterol precursors, such as 7-dehydrocholesterol (7-DHC), zymostenol and lathosterol [10, 11]. In particular, lathosterol is the penultimate cholesterol precursor and it has been found in plasma and tissues of patients with Lathosterolosis (OMIM #607330), firstly described by Brunetti-Pierri and co-workers [12]. The Lathosterolosis is a very rare autosomal recessive disorder caused by mutations in the gene encoding 3β-hydroxysteroid-Δ5-desaturase (EC 1.3.3.2). It is reported in only four cases [13] and the first live-born patient presented with a complex clinical phenotype, including multiple congenital anomalies, mental retardation and liver diseases, overlapping the Smith-Lemli-Opitz syndrome (SLOS, OMIM #270400), the prototype of inherited defects of cholesterol [14].

The early diagnosis of treatable cholesterol metabolism disorders, among which CTX and Lathosterolosis, is important because specific therapeutic treatment could prevent the disease progression [11, 13, 15]. The biochemical diagnosis of these defects is generally performed by analyzing sterol profile in the patient’s plasma and tissues by gas chromatography coupled with a flame ionization detector (GC-FID) and/or gas chromatography coupled to mass spectrometry (GC-MS) [11, 12].

The age of the patients suspected of having these disorders is highly variable due to the very different phenotypes. As age-related levels of these sterols are lacking, this study aims to determine and to compare preliminary plasma levels of cholestanol and lathosterol in unaffected newborns, children and healthy adults. To this aim, we analyzed overall the sterol profiles of 130 plasma samples by our previously standardized GC-FID method.

## Materials and methods

### Specimens

Newborn (age < 1 year; *n* = 24) and children plasma samples (*n* = 33) were selected retrospectively among those accepted in our laboratory for the diagnosis of cholesterol metabolism defects by GC-FID analysis of plasma sterol profile. We selected the samples that showed normal sterol profiles and were coming from patients without highly suggestive clinical phenotypes. In addition, anonymous adult plasma samples from apparently healthy blood donors (*n* = 73) have been provided from our Department of Transfusion Medicine. For these subjects the absence of cholestasis and liver diseases were excluded based on the evidence of normal levels of alkaline phosphatase, gamma-glutamyl-transferase, total bilirubin, total protein, albumin and aspartate and alanine transaminase.

### Sterols analysis

The analysis of plasma sterols was performed by GC-FID method as previously described [14, 16, 17]. Briefly, plasma (50 μL) was mixed with 100 μL of 5α-cholestane (IS, 0.4 mg/mL) and hydrolyzed using alkaline ethanol solution. The sterols were extracted with hexane and the dried residue was derivatized using a mixture of BSTFA and pyridine (7:3; v/v) to obtain trimethylsilyl derivatives. Then, the sterols were analyzed by a GC–FID system (HP-5890, Agilent Technologies, Santa Clara, CA, USA), equipped with a SAC-5 capillary column (Supelco, Taufkirchen, Germany).

### Statistical analysis

Continuous data were reported as mean ± standard deviation (SD) for parametric distributions or median and interquartile range for non-parametric distributions. Kolmogorov-Smirnov test was used to verify the normality of distributions. Categorical data were reported as frequency and percentage. Statistical differences among the three groups were assessed by ANOVA or non-parametric Kruskal-Wallis test. Comparisons between two groups were performed by T-test or non-parametric Mann-Whitney test as appropriate. To evaluate the correlation between variables the Spearman coefficient (sc) was calculated. The significance was accepted at the level of *p* <  0.05. For multiple comparisons of non-parametric distributions (cholestanol, cholestanol/cholesterol and cholestanol/lathosterol ratios) the Bonferroni correction was applied, i.e. considering a total α error = 0.05, a significance of 0.05/3 comparisons (*p* <  0.0167) was considered significant.

The software PASW version 18.0 software (SPSS Inc., Chicago, IL, USA) was used to perform statistical analyses.

## Results

**Fig. 1:**
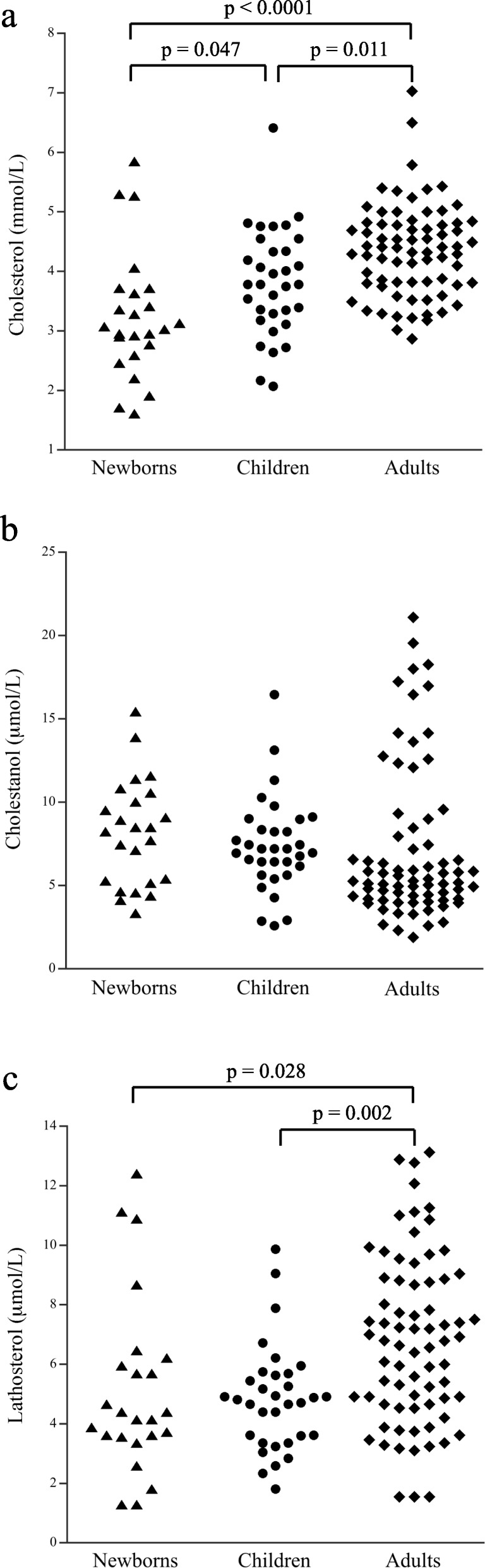
Distribution of cholesterol, cholestanol and lathosterol levels in newborns, children and adults. **a**: Dot plot of cholesterol levels; **b**: Dot plot of cholestanol levels); **c**: Dot plot of lathosterol levels. Statistical significances observed at multiple comparisons between groups are reported

The data of plasma cholesterol, cholestanol, lathosterol, cholestanol/cholesterol, lathosterol/cholesterol and cholestanol/lathosterol ratios obtained in newborns, children and adults are reported in Table 1. Statistically significant differences were observed among the three groups for all parameters. As expected, increase of cholesterol levels was observed from newborns to children and to adults (Fig. 1a). In addition, in all these groups, few outliers with high cholesterol levels were observed. In particular, in our preliminary data, there are three newborns with plasma cholesterol of 5.27, 5.30 and 5.85 mmol/L, one child with 6.41 mmol/L and three adults with 5.79, 6.50 and 7.03 mmol/L.

**Table 1:** Characteristics and sterol profiles of studied subjects

Parameters	Newborns(*n* = 24)	Children(*n* = 33)	Adults (*n* = 73)	Multiple comparisons (p)			
				Overall significance	Newborns vs Children	Newborns vs Adults	Children vs Adults
Gender (n° males and %)	18 (75.0%)	18 (54.5%)	51 (69.9%)	ns	nc	nc	nc
Age (years) ^a^	0.25 (0.08–0.54)	4.00 (2.75–6.96)	34 (26.5–41.0)	< 0.0001	nc	nc	nc
Cholesterol (mmol/L)	3.24 (1.06)	3.81 (0.90)	4.35 (0.78)	< 0.0001	0.047	< 0.0001	0.011
Cholestanol (μmol/L) ^a^	8.36 (5.18–10.42)	7.20 (5.90–8.67)	5.29 (4.21–8.21)	0.024	ns	ns	ns
Lathosterol (μmol/L)	5.15 (2.97)	4.83 (1.78)	6.78 (2.81)	0.0006	ns	0.028	0.002
Cholestanol/Cholesterol (μmol/mmol) ^a^	2.46 (1.76–3.54)	1.98 (1.49–2.26)	1.34 (1.06–1.90)	< 0.0001	0.011	0.0001	0.002
Lathosterol/Cholesterol (μmol/mmol)	1.72 (1.05)	1.29 (0.40)	1.57 (0.62)	0.050	ns	ns	ns
Cholestanol/Lathosterol (μmol/μmol) ^a^	1.88 (1.20–2.40)	1.66 (1.02–2.18)	0.84 (0.59–1.53)	0.001	ns	0.001	0.001

^a^Data of non-parametric distributions are reported as median and interquartile range and the comparisons are performed by Kruskal-Wallis test

*ns* not significant difference

*nc* not calculated

Although an overall difference of cholestanol levels was observed among the groups (*p* = 0.024), differences were not observed by multiple comparisons (Fig. 1b). The cholestanol range observed in adults is wider than in the two other groups with a maximum value of 21.1 μmol/L. The cholestanol/cholesterol ratio has been also evaluated and the results showed a statistical difference among the newborns, children and adults (Table 1).

For the lathosterol values, an overall difference was observed among the three groups, but multiple comparisons showed significant differences only between adults and newborns or children (Fig. 1c). Lathosterol/cholesterol ratio showed borderline significant difference among the three groups, (Table 1). Instead, the cholestanol/lathosterol ratio was significantly different among the three groups and, as observed for lathosterol levels, multiple comparisons showed significant differences only between adults and newborns or children (Table 1).

In the overall studied population, a positive correlation was observed between cholesterol levels and both cholestanol (sc = 0.290, *p* = 0.001) and lathosterol levels (sc = 0.353, *p* <  0.0001). In adults, the cholesterol levels also correlate with both cholestanol (sc = 0.465, *p* <  0.0001) and lathosterol levels (sc = 0.336, *p* = 0.004). Instead, in children, a statistically significant correlation was observed only between cholesterol and cholestanol levels (sc = 0.573, *p* = 0.0005). The correlations among cholesterol, cholestanol and lathosterol in newborns were not significant. Interestingly, opposite correlations between cholestanol/cholesterol and lathosterol/cholesterol ratios were observed in newborns (sc = 0.428, *p* = 0.037) and adults (sc = − 0.293, *p* = 0.012).

## Discussion

High cholestanol and lathosterol plasma levels are associated to CTX and Lathosterolosis, respectively. It is noteworthy that a delay in CTX diagnosis is usually reported, since the onset of symptoms generally occurs during the childhood/adolescence (from 6 to 16 years old) and the diagnosis does not occur until adulthood (25–35 years old) when additional clinical complications appear [18, 19]. In Italy, the mean age of CTX diagnosis estimated from 2012 to 2014 was 22.6 years old and the oldest diagnosed patient was 64 years old (http://old.iss.it/publ/). An early measurement of cholestanol would improve the CTX diagnosis starting an early treatment to prevent clinical complications as well as it would allow to differentiate this disease from other dyslipidemias such as sitosterolemia and familial hypercholesterolemia [20, 21].

Few papers report possible normal ranges of cholestanol, mainly determined in a low or unspecified subject number [22–24]. Only two studies report data on a high subject number, although they lack of a real calculation of reference values stratified for age [25, 26]. Unfortunately, no data are reported about cholestanol and lathosterol levels in children. The main purpose of this study was to measure and to analyze differences of cholestanol and lathosterol levels together with their ratio to cholesterol in subjects with different ages, e.g., newborns, children and adults.

In this study, no differences were observed for plasma cholestanol levels among the three groups. In adults, we found a 99th percentile equal to 19.9 μmol/L and a maximum value of 21.1 μmol/L. Dayspring et al. found a 99th percentile of 15.6 μmol/L in 667,718 patients with a mean age of 56 ± 15 years [25]; Schoefer et al. found a 99th percentile of 13.9 μmol/L in adult subjects [26].

Cholestanol and lathosterol have been validated not only as biomarkers of cholesterol metabolism defects, but also as surrogate markers for the evaluation of cholesterol homeostasis. We observed that the cholestanol/lathosterol ratio, which represents the cholesterol absorption/synthesis ratio [27], resulted significantly higher in both newborns and children than in adults. These results can be explained by the high cholesterol absorption in early age needed for body growth. On the other hand, we found that the plasma levels of lathosterol, a surrogate marker of cholesterol biosynthesis [27], were significantly lower in newborn and children than adults, whereas no differences are observed between newborns and children. Moreover, the correlation between cholestanol/cholesterol and lathosterol/cholesterol ratios was positive in newborns and negative in adults. These data further highlight a low cholesterol biosynthesis or an increased peripheral clearance of cholesterol and other sterols in newborns/children. The age-dependent increase of cholesterol synthesis and decrease of absorption agrees with the literature [28].

It is well known that increased cholestanol concentrations can also be found in patients with cholestasis or suffering from various liver diseases [29]. Therefore, in these cases the serum/plasma cholestanol concentrations do not reflect cholesterol absorption but its biliary excretion [30–32]. It is reported that, in end-stage cholestasis, serum cholestanol concentrations could increase to levels similar to those observed in CTX patients [33], though the cholestanol values observed in cholestatic conditions are lower than those observed in CTX patients as reported in several patients living in different countries [19, 23, 34]. All that highlights the importance of evaluating increased cholestanol concentrations together with carefully examining the patient’s clinical status. However, the patients with cholestasis and liver disease were excluded from groups here studied.

Cholestanol and lathosterol as well as other sterol surrogate markers offer effective data [35], and their analysis has been used in various studies to assess cholesterol metabolism in celiac disease, Crohn’s disease, cystic fibrosis, and other human diseases [16, 36, 37]. An additional evidence of cholestanol role in different diseases was reported by Civeira and colleagues that observed an increased prevalence of tendon xanthomas in genetically diagnosed Familial hypercholesterolemia (FH) patients with high levels of cholestanol [38]. The measurement of cholestanol could be useful as prognostic evaluation of tendon xanthoma development in adult and pediatric FH patients [39–42].

## Conclusions

In this study we reported that plasma levels of cholestanol and lathosterol and their ratios with cholesterol and lathosterol are significantly different among infants, children and adults. Since the two markers studied are useful for the diagnosis of CTX and Lathosterolosis, our results may indicate that the reference intervals should be differentiated for adults and children. Moreover, the molar ratio of both cholestanol/cholesterol and cholestanol/lathosterol discriminated the adult population from both children and newborns. Unfortunately, considering the low number of subjects analyzed in this work, we consider this study as an explorative research and we believe that further studies should be carried out on a greater number of samples.

## Data Availability

The datasets used and/or analysed during the current study are available from the corresponding author on reasonable request.

## Abbreviations

7-DHC: 7-dehydrocholesterol
CTX: Cerebrotendinous Xanthomatosis
*CYP27A1*: Sterol-27-hydroxylase gene
GC-FID: Gas chromatography-flame ionization detector
GC-MS: Gas chromatography-mass spectrometry
SOLS: Smith-Lemli-Opitz syndrome
